# Cardiopulmonary Bypass and Cancer Dissemination: A Logical But Unlikely Association

**DOI:** 10.21470/1678-9741-2018-0600

**Published:** 2018

**Authors:** Domingo M. Braile, Paulo Roberto B. Évora

**Affiliations:** 1 Faculdade de Medicina de São José do Rio Preto (FAMERP), São José do Rio Preto, SP, Brazil and Universidade de Campinas (UNICAMP), Campinas, SP, Brazil.; 2 Faculdade de Medicina de Ribeirão Preto da Universidade de São Paulo (FMRP-USP), Ribeirão Preto, SP, Brazil

## BJCVS Highlight

Several series indicated that surgical stress suppresses the immune system and that the use of cardiopulmonary bypass (CPB), cardiac arrest and ischemia contributes to this^[1,2]^. The most significant contributors to immune suppression are still yet to be determined. It seems that the survival of cancer patients who undergo cardiac surgery is more closely related to the progression of the tumor than the surgical procedure^[3]^. The reported results show a non-statistically significant increase in the risk of cancer-specific mortality for patients who underwent CPB before any diagnosis of cancer. In addition, a non-statistically significant increase was observed in the case-fatality rate for those cancer patients who underwent CPB surgery before the cancer diagnosis, and no difference was found in cancer stage at the time of diagnosis, with no impact on the relative risk of mortality. Further research is needed to know whether the transient immunosuppression associated with CPB can promote the spread and growth of pre-existing cancer cells, emphasizing that lung cancer and skin melanoma have had the most extensive association studies. In conclusion, adverse effects of CPB on cancer prognosis are expected but have not been confirmed. This editorial was motivated by the experience of an isolated case of an emphysematous, chronic smoker patient, who presented an impressive spread of an oat cell lung tumor 20 to 30 days after myocardial revascularization.

After our traditional "highlight", we recommend an attentive reading of the editorial by Prof. Rui Almeida that marks the beginning of his presidency at the Sociedade Brasileira de Cirurgia Cardiovascular (Brazilian Society of Cardiovascular Surgery). In addition, a second exciting editorial, written by Professors Luciano Albuquerque and Walter Gomes, who give us in this edition of the Brazilian Journal of Cardiovascular Surgery (BJCVS), a magnificent analytical study about the ORBITA trial that deserves the full attention of our readers.

Prof. Rui's reflections on training new generations of cardiac surgeons is a top priority for the future of our specialty, beginning with scientific initiation, medical residency, and class defense. It should be stressed that this has been a concern throughout the world, so we must join forces in the direction of these goals. Sure enough, BJCVS is a potent weapon for this purpose.

The ORBITA trial, recently published in The Lancet journal, brought important responses concerning possible placebo effect in stable angina treated by percutaneous intervention. Considering the ORBITA study as the best-designed trial comparing conservative and interventional strategies in patients with stable angina, doctors Albuquerque and Gomes, based on the rigor of the design trial, emphasize that percutaneous coronary intervention has a powerful placebo effect and should have an impact in forthcomings guidelines of stable coronary disease.

## Articles in this Issue

This issue of BJCVS presents a blind peer-reviewed selection of 16 articles that will surely please your readers:

- Two papers concerning on congenital heart disease presents and discuss: 1) the frequency of infective endocarditis in the Candida bloodstream in a children's hospital; 2) an unusual case of abdominal pain in childhood caused by a cardiac angiosarcoma.
- One presentation on risk factors applying an external validation of the European System for Cardiac Operative Risk Evaluation (EuroSCORE II) for risk prioritization in an Iranian population.
- One article on cardiac electrical stimulation presenting a survival analysis of chronic chagasic cardiomyopathy in patients under cardiac resynchronization therapy.
- Three articles on outcomes considering: 1) the experience of one Brazilian healthcare center with transcatheter aortic valve replacement; 2) long-term results of mitral valve repair; and 3) predictors of mortality in 10 years follow-up of surgical treatment of active infective endocarditis.
- One case report of a symptomatic aortic paravalvular leak: percutaneous treatment with the Amplatzer Vascular Plug III device as an alternative to surgery.
- One experimental paper evaluating the effects of atorvastatin and ischemic postconditioning in the prevention of ischemia and reperfusion injury.
- Three perioperative studies: 1) comparing the efficacy of selenium, vitamin C and n-acetylcysteine in the prevention of acute kidney injury following off-pump coronary artery bypass graft (OPCABG) surgery; 2) an analysis whether the preoperative use of beta-blockers in coronary artery bypass graft (CABG) patients with stable angina is associated to better cardiovascular survival; and 3) observations about postoperative bleeding following preoperative clopidogrel administration in patients with hemoglobin level above 110 g/L undergoing urgent CABG.
- One paper presenting the Freeman experience on early clinical results of the Perceval sutureless aortic valve in 139 patients.
- One paper asking if OPCABG for moderate chronic ischemic mitral regurgitation in the elderly is a superior option.
- One comparison between roller and centrifugal pump systems concerning hemolysis and inflammatory response to extracorporeal circulation during on-pump CABG.
- One systematic review about percutaneous lead extraction in infection of cardiac implantable electronic devices.

## References

[1] Suzuki S, Usui A, Yoshida K, Matsuura A, Ichihara T, Ueda Y (2010). Effect of cardiopulmonary bypass on cancer
prognosis. Asian Cardiovasc Thorac Ann.

[2] Pinto CA, Marcella S, August DA, Holland B, Kostis JB, Demissie K (2013). Cardiopulmonary bypass has a modest association with cancer
progression: a retrospective cohort study. BMC Cancer.

[3] Carrascal Y, Gualis J, Arévalo A, Fulquet E, Flórez S, Rey J (2008). Cardiac surgery with extracorporeal circulation in cancer
patients: influence on surgical morbidity and mortality and on
survival. Rev Esp Cardiol.

